# Protective Role of Methanol Leaf Extract of Catharanthus Roseus in Lipid Profile Modulation in Diabetic Wistar Rats

**DOI:** 10.7759/cureus.77420

**Published:** 2025-01-14

**Authors:** Balida Mallikarjuna Rao, T. Vedavijaya, Y. Roja Ramani, Suresh Babu Sayana

**Affiliations:** 1 Pharmacology and Therapeutics, Meenakshi Academy of Higher education and Research, Chennai, IND; 2 Department of Pharmacology, Meenakshi Ammal Dental College and Hospital, Chennai, IND; 3 Clinical Pharmacology, Orissa University of Health Sciences, Berhampur, IND; 4 Department of Pharmacology, Government Medical College and General Hospital, Bhadradri Kothagudem, IND

**Keywords:** catharanthus roseus, diabetes, hdl, ldl, lipid profile, total cholesterol, triglycerides, vldl, wistar rats

## Abstract

Background

Diabetes mellitus (DM) is a long-term metabolic condition frequently linked to lipid imbalances, which may elevate the risk of cardiovascular complications. Abnormalities in lipid profiles, such as increased cholesterol and triglycerides and decreased high-density lipoproteins (HDL), are commonly observed in diabetic individuals. Natural plant extracts, including *Catharanthus roseus*, have long been recognized for their medicinal properties, although their potential impact on lipid profiles requires further investigation.

Objective

To evaluate the effects of methanol leaf extract of *Catharanthus roseus* on the lipid profile of streptozotocin (STZ)-induced diabetic Wistar rats.

Methods

The study involved 30 male Wistar albino rats, divided into five groups: Normal Control (NC), Diabetic Control (DC), Positive Control (PC), Low Dose (LD-200 mg/kg) *Catharanthus roseus* (LD-200 mg/kg), and High Dose (HD-400 mg/kg) *Catharanthus roseus*. Diabetes was induced with a single intraperitoneal dose of streptozotocin (55 mg/kg). Animals with fasting blood glucose levels above 180 mg/dL were included. Treatments were administered for 30 days, with lipid profiles like total cholesterol, triglycerides, HDL, low-density lipoproteins (LDL), and very low-density lipoproteins (VLDL) assessed on the 15th and 30th days.

Results

Diabetic rats exhibited increased total cholesterol, triglycerides, LDL, and VLDL levels, and decreased HDL levels. Treatment with *Catharanthus roseus* extract significantly improved lipid profiles in a dose-dependent manner, with the HD-400 mg/kg showing the most notable changes, including increased HDL levels.

Conclusion

The study highlights the potential of *Catharanthus roseus* methanol leaf extract in improving lipid profiles in diabetic rats, suggesting its utility as a complementary therapy for managing dyslipidemia in diabetes.

## Introduction

Diabetes mellitus (DM) is a chronic metabolic disorder characterized by persistently elevated blood sugar levels due to insufficient insulin secretion and reduced insulin sensitivity [1]. Dyslipidemia, or abnormal lipid metabolism, is a frequent complication of diabetes. This condition is identified by high levels of total cholesterol, triglycerides, low-density lipoproteins (LDL), very low-density lipoproteins (VLDL), and low levels of high-density lipoproteins (HDL) [2]. Disruptions of the lipid profile to these extremes enormously increase the likelihood of cardiovascular disease, the largest cause of death and suffering from diabetes mellitus [3].

Managing the lipid profile is a very important component of diabetes care. Statins and fibrates are included in conventional pharmacological treatments of dyslipidemia [4]. However, these medications can have side effects or show limited efficacy in some patients; these natural products became popular as alternatives or for complementary therapy, which has led to an increasing interest in certain medicinal plants [5]. Lipid metabolism may be regulated by natural plant extracts, such as *Catharanthus roseus*, *Trigonella foenum-graecum* (fenugreek), and *Azadirachta indica* (neem), which have demonstrated safety and efficacy in managing diabetes-associated complications [6].

Folk medicine encompasses traditional healing practices that utilize natural remedies, including plant extracts like *Catharanthus roseus*. In India, such plants have been used for centuries, especially in rural communities, based on ancestral knowledge of their hypoglycemic, antioxidant, and anti-inflammatory properties for managing ailments like diabetes and related complications [7,8]. While glycemic control can contribute to lipid profile improvements, evidence suggests *Catharanthus roseus* exhibits direct lipid-modulating effects independent of its hypoglycemic properties. These effects are attributed to bioactive compounds such as flavonoids and alkaloids, which regulate lipid metabolism. This highlights the potential dual benefits of the plant extract in managing diabetes and dyslipidemia. Preliminary studies have also revealed that *Catharanthus roseus* may have an agreeable effect on lipid metabolism and hence, be considered a potential value in dyslipidemia during diabetes [8]. Scientific validation of its lipid-modulating properties is, however, limited, particularly in the experimental diabetic model.

This study aimed to evaluate the protective efficacy of the methanolic leaf extract of *Catharanthus roseus* in improving lipid profiles in streptozotocin (STZ)-induced diabetic Wistar rats. The research focused on exploring the therapeutic potential of *Catharanthus roseus* for managing dyslipidemia and preventing cardiovascular complications associated with diabetes by analyzing key lipid markers, including total cholesterol, triglycerides, HDL, LDL, and VLDL.

## Materials and methods

Study design and duration

The work was conducted at the Roland Institute of Pharmaceutical Sciences, Berhampur, between the month of February 2023 and the month of February 2024. The effect of the methanolic leaf extract of *Catharanthus roseus* on lipid profile modulation in Wistar albino rats with STZ-induced diabetes was evaluated using a randomized controlled experimental design.

Animals

Thirty male Wistar albino rats weighing between 200 and 250 g were used. All rats were housed under standard laboratory conditions, including a 12-hour light/dark cycle with free access to standard pellet food and water. One week was spent acclimatizing the animals to the environment prior to the experiment beginning.

The sample size of 30 rats was determined using the "resource equation method," a widely used approach in preclinical studies to ensure statistically significant results while adhering to ethical guidelines for minimizing animal use. This sample size allows for adequate replication across five experimental groups, ensuring reliable comparisons and reproducible outcomes. Additionally, the study design and sample size were guided by previous research investigating the effects of plant-based extracts on lipid profiles in diabetic rats, which employed similar group sizes to achieve robust conclusions [9].

Only male Wistar rats were included in the study to avoid potential variability due to hormonal fluctuations in females, which could influence lipid metabolism and glycemic control. This approach ensures consistency in the results, minimizing confounding factors and enhancing the reliability of the findings [10].

Induction of diabetes

STZ was administered at a single dose of 55 mg/kg body weight intraperitoneally, dissolved in 100 mmol of citrate buffer (pH 4.0-4.5). Nicotinamide at a dose of 120 mg/kg body weight was administered 40 minutes before STZ injection to minimize mortality. Nicotinamide was used in the study to partially protect pancreatic beta cells from the cytotoxic effects of STZ, thereby inducing moderate hyperglycemia that closely mimics the pathophysiology of Type 2 diabetes (T2D). This approach prevents complete beta-cell destruction, which is characteristic of Type 1 diabetes, and allows for the establishment of a diabetic model suitable for evaluating the lipid-modulating effects of *Catharanthus roseus*. By using nicotinamide, the study ensures a more accurate representation of T2D, facilitating relevant observations on the therapeutic potential of the plant extract. STZ selectively destroys and targets insulin‐producing beta cells in the pancreas, inducing hyperglycemia [11,12]. Rats with fasting blood glucose levels ≥180 mg/dL, measured with a glucometer, were selected after 48 hrs for this study.

Grouping and treatment

The study involved 30 rats, which were randomly divided into five groups, each consisting of six rats. Group 1 served as the normal control (NC) and comprised non-diabetic rats receiving no treatment. Group 2, the diabetic control (DC), included diabetic rats that received no treatment. Group 3 consisted of diabetic rats treated with a low dose (LD-200 mg/kg) of *Catharanthus roseus* leaf extract, while Group 4 included diabetic rats treated with a high dose (HD-400 mg/kg) of the same extract. Group 5, the positive control (PC), comprised diabetic rats receiving standard lipid-lowering therapy. The *Catharanthus roseus* leaf extract was administered orally to the rats via gavage once daily for 30 days, starting 48 hours after the confirmation of diabetes. This method ensured precise and consistent dosing, independent of the animals' feeding behaviors, thereby minimizing variability and enhancing the reliability of results. The 30-day treatment duration was selected to allow sufficient time for metabolic changes and measurable effects on lipid profiles to develop, aligning with standard protocols for evaluating plant-based interventions while adhering to ethical guidelines for animal research. 

The methanolic extract of *Catharanthus roseus* was prepared by collecting fresh leaves, which were thoroughly washed, air-dried in the shade, and powdered using a mechanical grinder. A measured quantity of the leaf powder was subjected to maceration in methanol (1:10 w/v) for 48 hours at room temperature with occasional stirring to ensure maximum extraction of bioactive compounds. The mixture was then filtered through Whatman No. 1 filter paper to remove plant debris. The filtrate was concentrated under reduced pressure using a rotary evaporator at 40°C to remove the methanol, yielding a semi-solid crude extract. The extract was stored in an airtight container at 4°C until further use in the study [7,8].

Sample collection and lipid profile evaluation

Blood samples were collected from the retro-orbital plexus under mild anesthesia on Day 15 and Day 30 of the study. Adverse effects were not formally screened on Day 15, as the study primarily focused on evaluating the cumulative lipid-modulating effects of *Catharanthus roseus* over 30 days. However, interim data collection on Day 15 was conducted to monitor the progression of lipid profile changes, and no visible signs of toxicity or abnormal behavior were observed in the animals during this period. The collected samples were centrifuged to separate serum for lipid profile analysis. The parameters total cholesterol, triglycerides, HDL, LDL, and VLDL were selected as they are standard markers for evaluating lipid profile abnormalities associated with diabetes. These markers provide a comprehensive understanding of lipid metabolism and cardiovascular risk. These biochemical parameters were assessed using standard enzymatic kits, following the manufacturer’s protocols.

Statistical analysis

Data are presented as mean ± standard deviation (SD). Statistical comparisons between groups were made using one-way analysis of variance (ANOVA) with a post hoc Tukey's test. A p-value of <0.05 was interpreted as statistically significant. Statistical analyses were performed using IBM Statistical Package for the Social Sciences (SPSS) statistical software, version 26 (IBM Corp., Armonk, NY, USA). This software is widely utilized for statistical analysis in research due to its robust capabilities for data management, advanced statistical testing, and graphical representation of results.

Ethical considerations

The care and use of laboratory animals conformed to ethical guidelines. The experimental protocols were approved by the Institutional Animal Ethics Committee (IEC/RIPS/2022/159; dated 16/9/2022) of the Roland Institute of Pharmaceutical Sciences, Berhampur. An attempt was made to minimize animal suffering as much as possible.

## Results

Lipid profile modulation in diabetic Wistar rats

The lipid profile analysis revealed significant alterations in the DC group compared to the NC group at both 15 and 30 days. The total cholesterol levels in the DC group were significantly elevated at 15 days (248.4 ± 29.6 mg/dL) and further increased at 30 days (286.3 ± 27.5 mg/dL), compared to the NC group (140.8 ± 2.5 mg/dL and 143.3 ± 3.14 mg/dL at 15 and 30 days, respectively). Treatment with *Catharanthus roseus* demonstrated dose-dependent reductions in total cholesterol levels. In the LD-200 mg/kg group, cholesterol levels were reduced to 174.7 ± 7.4 mg/dL at 15 days and 259.7 ± 22.2 mg/dL at 30 days. The HD-400 mg/kg group showed more pronounced reductions, with cholesterol levels of 164.3 ± 7.3 mg/dL at 15 days and 179.4 ± 18.8 mg/dL at 30 days (p < 0.001). The PC group exhibited total cholesterol levels of 152.7 ± 5.4 mg/dL at 15 days and 151 ± 7.3 mg/dL at 30 days (Table 1).

**Table 1:** Total cholesterol and triglyceride levels in diabetic Wistar rats NC: Normal Control; DC: Diabetic Control; LD: Low Dose; HD: High Dose; PC: Positive Control Values are expressed as mean ± SD with corresponding p-values indicating statistical significance.

Group	Total Cholesterol - 15 Days (mg/dL)	p-value	Total Cholesterol - 30 Days (mg/dL)	p-value	Triglycerides - 15 Days (mg/dL)	p-value	Triglycerides - 30 Days (mg/dL)	p-value
NC	140.8 ± 2.5	<0.001	143.3 ± 3.14	<0.001	66.9 ± 4.9	<0.001	69.6 ± 5.2	<0.001
DC	248.4 ± 29.6	<0.001	286.3 ± 27.5	<0.001	158.4 ± 4.3	<0.001	167.7 ± 3.9	<0.001
LD-200 mg/kg	174.7 ± 7.4	<0.001	259.7 ± 22.2	<0.001	147.7 ± 7.3	<0.001	157.4 ± 7.6	<0.001
HD-400 mg/kg	164.3 ± 7.3	<0.001	179.4 ± 18.8	<0.001	121 ± 3.2	<0.001	128.4 ± 5.5	<0.001
PC	152.7 ± 5.4	<0.001	151 ± 7.3	<0.001	77.3 ± 3.1	<0.001	87.3 ± 3.1	<0.001

Triglyceride levels before and after the treatment of *Catharanthus roseus *in diabetic Wistar rats

Similarly, serum triglyceride levels were significantly elevated in the DC group, reaching 158.4 ± 4.3 mg/dL at 15 days and 167.7 ± 3.9 mg/dL at 30 days, compared to the NC group (66.9 ± 4.9 mg/dL and 69.6 ± 5.2 mg/dL at 15 and 30 days, respectively). *Catharanthus roseus* treatment resulted in a dose-dependent reduction in triglyceride levels. In the LD-200 mg/kg group, triglyceride levels decreased to 147.7 ± 7.3 mg/dL at 15 days and 157.4 ± 7.6 mg/dL at 30 days, while the HD-400 mg/kg group exhibited levels of 121 ± 3.2 mg/dL at 15 days and 128.4 ± 5.5 mg/dL at 30 days. The PC group had triglyceride levels of 77.3 ± 3.1 mg/dL at 15 days and 87.3 ± 3.1 mg/dL at 30 days (p < 0.001) (Table 1).

HDL levels before and after the treatment of *Catharanthus roseus *diabetic Wistar rats

HDL levels, which were reduced in the DC group (26.6 ± 1.3 mg/dL at 15 days and 20.8 ± 1.5 mg/dL at 30 days), improved following *Catharanthus roseus* treatment. The HD-400 mg/kg group showed HDL levels of 31 ± 0.9 mg/dL at 15 days and 32.7 ± 1.9 mg/dL at 30 days. In the LD-200 mg/kg group, HDL levels were 29.7 ± 0.5 mg/dL at 15 days and 31.7 ± 1.4 mg/dL at 30 days. The PC group had HDL levels of 32 ± 0.9 mg/dL at 15 days and 34.4 ± 1.4 mg/dL at 30 days (p < 0.001) (Table 2).

**Table 2:** HDL, LDL, and VLDL levels in diabetic Wistar rats NC: Normal Control; DC: Diabetic Control; LD: Low Dose; HD: High Dose; PC: Positive Control; HDL: High-density lipoproteins; LDL: Low-density lipoproteins; VLDL: Very low-density lipoproteins Values are expressed as mean ± SD with corresponding p-values indicating statistical significance.

Group	HDL - 15 Days (mg/dL)	p-value	HDL - 30 Days (mg/dL)	p-value	LDL - 15 Days (mg/dL)	p-value	LDL - 30 Days (mg/dL)	p-value	VLDL - 15 Days (mg/dL)	p-value	VLDL - 30 Days (mg/dL)	p-value
NC	26.6 ± 1.3	<0.001	20.8 ± 1.5	<0.001	90 ± 0.9	<0.001	91 ± 0.9	<0.001	19.3 ± 1.4	<0.001	21.7 ± 2.7	<0.001
DC	26.6 ± 1.3	<0.001	20.8 ± 1.5	<0.001	186.6 ± 2.5	<0.001	207.3 ± 2.4	<0.001	53.6 ± 6.1	<0.001	60.5 ± 3.3	<0.001
LD-200 mg/kg	29.7 ± 0.5	<0.001	31.7 ± 1.4	<0.001	166.4 ± 5.1	<0.001	186.4 ± 4	<0.001	49 ± 6.8	<0.001	53 ± 5	<0.001
HD-400 mg/kg	31 ± 0.9	<0.001	32.7 ± 1.9	<0.001	130.7 ± 9.4	<0.001	143 ± 5.6	<0.001	36.7 ± 4	<0.001	41.4 ± 5.4	<0.001
PC	32 ± 0.9	<0.001	34.4 ± 1.4	<0.001	110.4 ± 4.9	<0.001	113.7 ± 6.3	<0.001	25 ± 2.7	<0.001	26.7 ± 3.1	<0.001

LDL levels before and after the treatment of *Catharanthus roseus* diabetic Wistar rats

LDL levels were significantly higher in the DC group (186.6 ± 2.5 mg/dL at 15 days and 207.3 ± 2.4 mg/dL at 30 days) compared to the NC group (90 ± 0.9 mg/dL at 15 days and 91 ± 0.9 mg/dL at 30 days). *Catharanthus roseus* treatment significantly reduced LDL levels, with the HD-400 mg/kg group showing levels of 130.7 ± 9.4 mg/dL at 15 days and 143 ± 5.6 mg/dL at 30 days. The LD-200 mg/kg group showed levels of 166.4 ± 5.1 mg/dL at 15 days and 186.4 ± 4 mg/dL at 30 days, while the PC group had LDL levels of 110.4 ± 4.9 mg/dL at 15 days and 113.7 ± 6.3 mg/dL at 30 days (p < 0.001) (Table 2).

VLDL levels before and after the treatment of *Catharanthus roseus* diabetic Wistar rats

VLDL levels were also significantly elevated in the DC group (53.6 ± 6.1 mg/dL at 15 days and 60.5 ± 3.3 mg/dL at 30 days) compared to the NC group (19.3 ± 1.4 mg/dL and 21.7 ± 2.7 mg/dL at 15 and 30 days, respectively). *Catharanthus roseus* treatment significantly reduced VLDL levels, with the LD group showing levels of 49 ± 6.8 mg/dL at 15 days and 53 ± 5 mg/dL at 30 days, while the HD-400 mg/kg group had levels of 36.7 ± 4 mg/dL at 15 days and 41.4 ± 5.4 mg/dL at 30 days. The PC group exhibited VLDL levels of 25 ± 2.7 mg/dL at 15 days and 26.7 ± 3.1 mg/dL at 30 days (p < 0.001) (Table 2).

## Discussion

The effect of methanolic leaf extract of *Catharanthus roseus* on modulation of lipid profile was investigated using Wistar rats with STZ-induced hyperglycemic rats. The study showed that administration of *Catharanthus roseus* helped to normalize serum levels of total cholesterol, triglycerides, and LDL and VLDL, while increasing HDL in these rats. These results indicate that *Catharanthus roseus* may serve as a protective agent against diabetes-induced dyslipidemia and could potentially be used as an adjunct therapy for managing lipid abnormalities in diabetic patients.

The DC group exhibited notable elevations in total cholesterol, triglycerides, LDL, and VLDL levels, consistent with the dyslipidemic state commonly associated with diabetes. Dyslipidemia in diabetes results from elevated levels of unbound fatty acids, which promote the enhanced synthesis of triglycerides and VLDL, along with reduced HDL levels, thus enhancing the risk of cardiovascular diseases [13-15]. In the current research work, the administration of *Catharanthus roseus* leaf extract led to a concentration-dependent improvement in lipid profile parameters.

The high-dose *Catharanthus roseus* (HD-400 mg/kg) group demonstrated the most pronounced reduction in total cholesterol and triglyceride levels, nearing those observed in the normal control group. This effect is likely due to the bioactive compounds in *Catharanthus roseus*, including flavonoids and alkaloids, which are known for their hypolipidemic and antioxidant properties [16,17]. These compounds may inhibit cholesterol biosynthesis and improve lipid metabolism, thereby contributing to the observed decrease in serum lipid levels. 

Reverse cholesterol transport, a process that moves excess cholesterol from peripheral tissue to the liver via HDL, or 'good cholesterol,' is vital [18]. HDL levels were markedly elevated in both the LD-200 mg/kg and HD-400 mg/kg *Catharanthus roseus* treatment groups, especially the high-dose treatment group. Due to particularly important HDL's rise, it can contribute to lowering the cardiovascular risk in diabetic conditions [18,19].

The positive control group, which received standard lipid-lowering therapy, also showed improvements in lipid profile parameters, though *Catharanthus roseus* treatment, particularly at higher doses, demonstrated comparable and, in some cases, greater effects. This suggests that *Catharanthus roseus* could be an effective alternative or complementary therapy for managing dyslipidemia in diabetic patients, potentially offering fewer side effects than conventional drugs.

While the precise mechanisms of action of *Catharanthus roseus *remain to be fully elucidated, the observed lipid-lowering effects may be attributed to its antioxidant, anti-inflammatory, and hypolipidemic properties. The bioactive compounds in *Catharanthus roseus* could potentially inhibit key enzymes involved in lipid biosynthesis, reduce oxidative stress, and improve insulin sensitivity, all of which are critical in the regulation of lipid metabolism in diabetic conditions [16,17].

Limitations

Although the findings are promising, the study is limited by its relatively short duration (30 days) and reliance on an animal model. Additionally, a detailed phytochemical analysis of *Catharanthus roseus* is required to identify the specific phytochemical compounds responsible for its lipid-modulating effects.

## Conclusions

This study highlights the significant lipid-modulating effects of methanol leaf extract of *Catharanthus roseus* in STZ-induced diabetic Wistar rats. The treatment led to dose-dependent reductions in total cholesterol, triglycerides, LDL, and VLDL levels, with a notable increase in HDL levels. Among the groups, the HD-400 mg/kg dose showed the most pronounced effects, bringing lipid profile parameters closer to normal levels. These findings emphasize the hypolipidemic potential of *Catharanthus roseus*, suggesting its value as a natural therapeutic option for managing diabetes-associated dyslipidemia and reducing cardiovascular risk.

Additionally, the results suggest the plant's potential as an adjunct therapy for comprehensive diabetes management. However, further studies are needed to explore the long-term efficacy and safety of *Catharanthus roseus*.
